# The PI3K p110δ Regulates Expression of CD38 on Regulatory T Cells

**DOI:** 10.1371/journal.pone.0017359

**Published:** 2011-03-01

**Authors:** Daniel T. Patton, Marcus D. Wilson, Wendy C. Rowan, Dalya R. Soond, Klaus Okkenhaug

**Affiliations:** 1 Laboratory of Lymphocyte Signalling and Development, Babraham Institute, Cambridge, United Kingdom; 2 Tool Monoclonal Antibody Group, GlaxoSmithKline Research and Development, Stevenage, United Kingdom; Centre de Recherche Public de la Santé (CRP-Santé), Luxembourg

## Abstract

The PI3K pathway has emerged as a key regulator of regulatory T cell (Treg) development and homeostasis and is required for full Treg-mediated suppression. To identify new genes involved in PI3K-dependent suppression, we compared the transcriptome of WT and p110δ^D910A^ Tregs. Among the genes that were differentially expressed was the gene for the transmembrane cyclic ADP ribose hydrolase CD38. Here we show that CD38 is expressed mainly by a subset of Foxp3^+^CD25^+^CD4^+^ T cells originating in the thymus and on Tregs in the spleen. CD38^high^ WT Tregs showed superior suppressive activity to CD38^low^ Tregs, which failed to upregulate CD73, a surface protein which is important for suppression. However, Tregs from heterozygous CD38^+/−^ mice were unimpaired despite lower levels of CD38 expression. Therefore, CD38 can be used as a marker for Tregs with high suppressive activity and the impaired Treg function in p110δ^D910A^ mice can in part be explained by the failure of CD38^high^ cells to develop.

## Introduction

The role of regulatory T cells (Tregs) in preventing systemic autoimmunity and to limit inflammation is well established. CD4^+^Foxp3^+^ T which develop from CD4^+^CD8^+^ T cell precursors in the thymus are referred to as natural Tregs [1], [2]. Induced Tregs develop from Foxp3^−^CD4^+^ T cells in the peripheral immune organs in presence of low concentrations of antigen or TGF-β [3], [4], [5], [6]. Tregs play a critical role in limiting the responses of not only other T cells, but also B cells and components of the innate immune system to antigen and/or inflammatory stimuli. Several mechanisms have been proposed as to how Treg function [7]. The expression of CTLA-4 is essential for Treg function by a mechanism thought to involve the suppression of APCs [8], [9]. Tregs also express high levels of CD25 which may consume available IL-2 thus depriving T helper cells of this cytokine [10]. CD39 and CD73 expressed by Tregs generate adenosine which has an immunosuppressive effect on Th cells [11]. Tregs also mediate immunosuppression in different physiological contexts by secreting the anti-inflammatory cytokines including IL-10, IL-35 and TGF-β [9], [10], [12], [13], [14], [15], [16].

The Class I PI3K enzymes phosphorylate the D3-position of Phosphatidylinositol PtdIns(4,5)P_2_ to generate PtdIns(3,4,5)P_3_ which in turn is bound by proteins such as Pdk1, Akt and Itk that contain a pleckstrin homology domain [17]. Four catalytic isoforms of Class I PI3K are expressed in T cells: p110α, p110β, p110γ and p110δ [17]. p110α, p110β and p110δ form heterodimers with SH2-domain containing p85, p55 or p50 regulatory subunits whereas p110γ is bound by a p101 or p84 regulatory subunit. In T cells antigen, costimulatory and cytokine receptors activate p110δ, whereas p110γ is activated by chemokine receptors [18]. We have previously shown that Treg development, differentiation and function are altered in p110δ^D910A^ mice which possess a kinase-dead mutant of p110δ [19]. Treg development in the thymus was enhanced whereas there were fewer Tregs in the peripheral organs. Importantly, p110δ^D910A^ Tregs were impaired in their capacity to suppress the proliferation of responder CD4^+^ T cells, secreted reduced levels of IL-10 and failed to suppress inflammation of the colon [19]. Moreover, p110δ^D910A^ mice were resistant to infection by *Leishmania major* and this was attributed to defective Treg expansion and recruitment to the site of infection [20]. However, despite their impaired function, p110δ^D910A^ Tregs express similar levels of Foxp3, CD25 and CTLA-4 [19]. Since IL-10 is not essential for all Treg-dependent functions, this leaves open the question of the precise nature of the suppressive mechanism that is defective in p110δ^D910A^ Tregs. Deletion of the p85α and p85β PI3K regulatory isoforms in T cells resulted in a reduction in Tregs in the spleen and development of a Sjogren's-syndrome-like disease; however, whether this is linked to Treg-deficiency has not been determined [21]. More recently, Pdk1 has been shown to be essential for Treg function, but not for Treg development, which is consistent with this being and important signaling protein downstream of p110δ [22].

The role of PI3Ks in Treg development and function has been further emphasized by the identification of Foxo transcription factor binding sites in the Foxp3 promoter and by the observation that Treg development is impaired in mice with a T cell-specific deletion of Foxo1 and Foxo3 [23], [24], [25]. PI3Ks regulate Foxo activity via Akt, which phosphorylates Foxo proteins leading to their sequestration in the cytoplasm [26]. Therefore, inhibition of PI3K could lead to enhanced Foxo-dependent Foxp3 expression and may help explain why there are more Foxp3 cells in the thymus of p110δ-deficient mice. Whether Foxo plays a similar role in peripheral Treg is less clear given the reduced proportions of Treg in the spleen and lymph nodes of p110δ^D910A^ mice. Moreover, CD4^Cre^-Foxo1 mice had more Tregs in the peripheral organs, despite reduced proportions in the thymus. The serine/threonine kinase mTOR integrates signals for the PI3K and MAP-kinase pathways in T cells [27]. Surprisingly, the mTOR inhibitor rapamycin enhances differentiation of Treg cells by a mechanism that has yet to be fully understood, but which may involve Foxp3-dependent upregulation of Pim2 [28], [29], [30]. Hence, the PI3K pathway can affect Treg numbers positively or negatively, depending in part on their stage of development and anatomical context.

To gain a more complete understanding of the role of p110δ-dependent transcriptional regulation in Treg development and function we compared the transcriptome of p110δ^D910A^ Treg with WT Treg, and found reduced expression of the gene for the transmembrane cyclic ADP ribose hydrolase CD38. Sorted CD38^high^ Treg showed superior suppressive capacity to CD38^low^ Treg. However, CD38^+/−^ heterozygous Treg showed normal suppression in vitro suggesting that reduced CD38 expression per se is insufficient to abolish Treg activity, but rather identifies CD38^high^ Tregs as a population of highly suppressive Treg that fails to develop in p110δ^D910A^ mice.

## Methods

### Mice

CD38^−/−^, p110δ^D910A^, and RAG2^−/−^ have been previously described [31], [32], [33] and were maintained on the C57BL/6 (B6) background. Congenic B6.SJL mice (in which the CD45.1 allele from the SJL strain has been backcrossed onto the B6 genetic background) were originally purchased from Taconic. All experimental protocols had been approved by the UK Home Office and local ethical review (PPL 80/1809 and 80/2248).

### Reagents

The following antibodies were purchased from eBioscience or Becton Dickinson: CD4 (GK1.5), CD8 (53-5.7), CD25 (PC61.5), CD38 (clone 90), CD45.1 (A20), CD45.2 (clone 104), CD73-PE, CD90.2 (53.2.1), CTLA4 (UCH10-4B9), Foxp3 (FJK-16s), GITR (DTA-1), ICOS (15F9). Anti-CD3ε (clone 145-2C11) was prepared in-house.

### Microarray experiments

RNA from 2×10^6^ lymph node CD4^+^CD25^+^ cells from 6–8 mice was isolated using Trizol, biotinylated, fragmented and hybridised to Affymetrix GeneChip Mouse Genome 430 2.0 mouse arrays according to the manufacturer's protocols (array service provided by Geneservice). Three separate preparations of RNA from each genotype were compared using GeneSpring (cut-off: 2 fold difference, p<0.01). Microarray data has been submitted to ArrayExpress with the accession number E-MEXP-2955.

### Quantitative real-time PCR (qRT-PCR)

cDNA was synthesized from RNA purified as above using the Superscript II kit (Invitrogen). PCR was performed using SYBR green PCR mastermix (Applied Biosystems) and a Chromo4 machine (Bio-Rad). Primer sequences are given in Table S1.

### Bone Marrow Chimeras

RAG2^−/−^ mice were irradiated with 20 gy and reconstituted with 5×10^6^ cells from a 1∶1 mixture of either WT∶B6.SJL or p110δ^D910A^∶B6.SJL bone marrow. After eight weeks, the mice were dissected and spleen cells analyzed by FACS.

### Regulatory T cell purification and co-culture experiments

Tregs cells were purified using Miltenyi magnetic beads and by FACS to greater than 98% purity. CD4^+^CD25^−^ cells were purified by negative selection using magnetic beads. In some experiments, the CD4^+^CD25^−^ cells were stained using 2 µM CFSE (Molecular Probes) for seven minutes at room temperature. For APC preparations, splenocytes were depleted of T cells by labeling with an anti-Thy1.2 antibody (Sigma) and lysing the T cells using Rabbit LowTox-M complement (Cedarlane, Burlington, Ontario, Canada). The remaining cells were layered over Lympholyte-M (Cedarlane) and centrifuged, with the cells from the interface layer irradiated and used as APCs. 10^5^ CD25^−^ responder cells and 10^5^ APCs were added to wells of a 96 U-well plate along with 1µg/ml anti-CD3ε. CD25^+^ regulatory cells were added at ratios of 1-1 to 1-32 to the CD25^−^ cells. All experiments were performed in RPMI-1640 media (Invitrogen, Paisley, UK) containing 5% FCS (Biosera, Sussex, UK), 1% Penicillin-Streptomycin (Sigma) and 5×10^−5^ M 2-mercaptoethanol (Sigma). After three days the cells were labeled with anti-CD45.1-PE, anti-CD4-APC, anti-CD90.2-PE and anti-CD45.2 APC-eFluor780 and analyzed on a LSRII flow cytometer (BD) in a buffer containing 2 µg/ml 7-AAD (Molecular Probes). The division history of CD4^+^CD90.2^+^CD45.1^+^CD45.2^−^7AAD^−^ responder cells was analyzed using Flowjo v8.8.6 (TreeStar, Stanford, US) and the division index (mean divisions per divided cell) plotted. Alternatively, proliferation was measured by ^3^H-thymidine incorporation.

### Differentiation of Treg from CD4+CD25^−^ T cells

10^5^ CD4^+^CD25^−^ cells were placed in culture with 10^5^ APCs and stimulated with one or more of: 1µg/ml anti-CD3e (2C11), 20 ng/ml TGF-β1 (Peprotech or eBioscience), all-trans retinoic acid in ethanol (ATRA, Sigma) or with ethanol or DMSO at equivalent concentrations used as a vehicle controls. 3µM PI103 (pan-class I and mTOR inhibitor, Calbiochem) and 3µM IC87114 (p110δ selective inhibitor, synthesized in-house) were used to inhibit PI3K. After three days, the cells were stained with CD73-PE, Foxp3-Alexa647, CD38-FITC and analyzed by flow cytometry.

## Results

### CD38 is one of a limited number of differentially expressed transcripts in p110δ ^D910A^ Tregs

**Figure 1:**
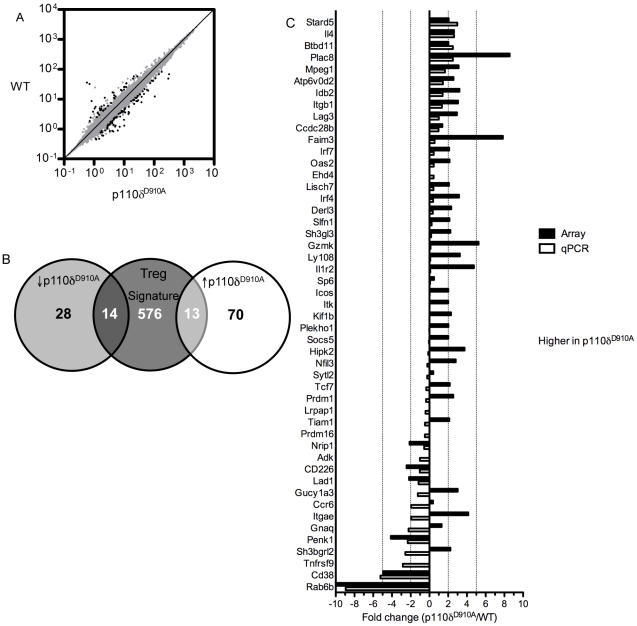
Genome-wide expression-profiling of WT and p110δ^D910A^ CD4^+^CD25^+^cells. CD4^+^CD25^+^ cells were isolated from WT and p110δ^D910A^ mice and RNA expression analysed by gene arrays. A. Shown is data from all 45,002 probe sets plotted as expression in WT Treg against expression in p110δ^D910A^ Treg (n = 3 for each genotype). Black dots represent genes that were significantly differentially expressed between WT and p110δ^D910A^ (greater than 2-fold difference and p<0.01). B. Differentially expressed genes from A were then compared against a previously published set of Treg signature genes [34]. The numbers of probe sets belonging in each section of the Venn diagram are shown. C. Comparison of expression data from selected probe sets found to be significantly different in A with qRT-PCR performed on RNA from separate preparations of WT and p110δ^D910A^ Treg.

We have previously shown that p110δ^D910A^ CD4^+^CD25^+^ cells are impaired in their ability to suppress the responses of conventional T cells [19]. To identify differentially expressed transcripts that may lead to this impairment, cDNA from p110δ^D910A^ and wild type (WT) Treg cells was hybridized to Affymetrix arrays. 125 out of 45,002 probe sets were significantly (p<0.01 and 2-fold different) different between WT and p110δ^D910A^ (Fig. 1A and Table S2). Of these, 27 belonged to a set of 603 Treg signature probe sets described by Hill *et al.* [34] (Fig. 1B). However, the expression of well-characterized Treg-associated genes, including Foxp3, CD25, CTLA-4 was unaltered, suggesting that the CD4^+^CD25^+^ T cells from p110δ^D910A^ mice genuinely belong to the Treg lineage (Fig. 1B).

We next performed qRT-PCR analysis of WT and p110δ^D910A^ Tregs. Although most genes showed a similar pattern of differential expression, only seven genes were confirmed to be expressed at two-fold higher or lower levels in p110δ^D910A^ Tregs compared to WT Tregs by this method (*Stard5*, *IL4*, *Btbd11*, *Plac8*, *Penk1*, *Cd38* and *Rab6b*) (Fig. 1C). As CD38 has been previously described as a marker of T cells with regulatory function [35] and because CD38^−/−^ mice bred to the NOD background are more susceptible to develop diabetes [36], we investigated its role in Treg biology and its regulation by PI3K.

**Figure 2:**
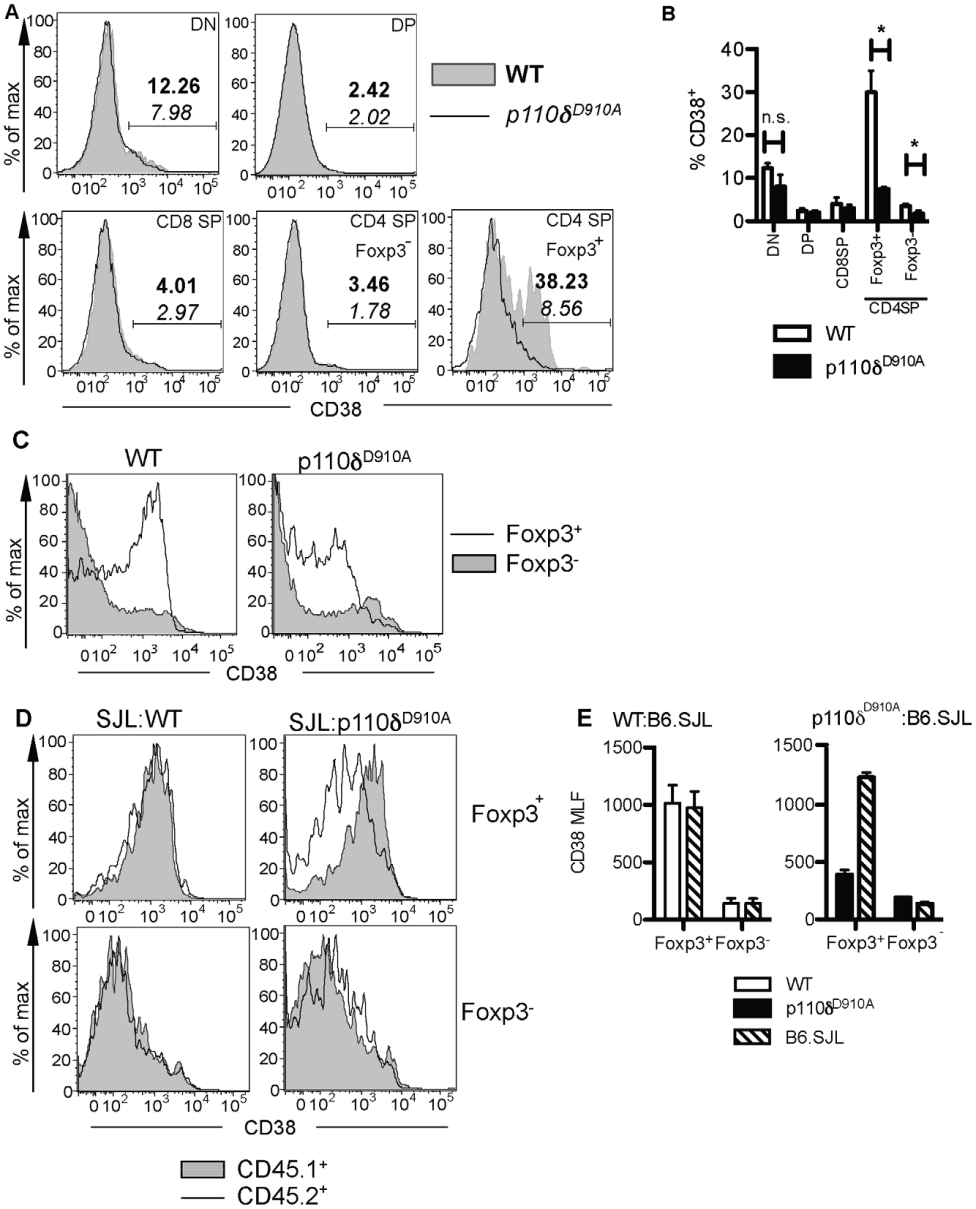
Expression of CD38 by WT and p110δ^D910A^ CD4^+^Foxp3^+^ Treg cells. A. Expression of CD38 on DN, DP and CD4 and CD8 single-positive cells from the thymus of WT and p110δ^D910A^ mice. Mean percentages of the cells within the CD38^+^ gate are for WT (**bold** typeface) and p110δ^D910A^ mice (*italic*). B. Summary of statistics shown in A (n = 3 for each genotype). C. Expression of CD38 on Foxp3^+^ and Foxp3^−^ cells from the spleen of WT and p110δ^D910A^ mice. D. Representative FACS plots of CD38 on Foxp3^+^CD4^+^ T cells from the spleens of WT∶B6.SJL or p110δ^D910A^∶B6.SJL bone marrow chimeras. E. Mean expression of CD38 on Foxp3^+^ and Foxp3^−^ cells from WT∶B6.SJL or p110δ^D910A^∶B6.SJL bone marrow chimeras, n = 4 for WT∶B6.SJL and n = 3 for p110δ^D910A^∶B6.SJL.

We first defined when CD38 was expressed during T cell development. Few CD4^−^CD8^−^ (double negative, DN), CD4^+^CD8^+^ (double positive, DP), CD8^+^CD4^−^ (CD8 single positive (CD8 SP) or CD4^+^CD8^−^Foxp3^−^ (CD4 SP Foxp3^−^) cells from the thymus of WT or p110δ^D910A^ mice expressed CD38 (Fig. 2A). However, high levels of CD38 were expressed on a proportion of WT CD4 SP Foxp3^+^ cells (Fig. 2A and 2B). In contrast, significantly fewer CD4 SP Foxp3^+^ cells from p110δ^D910A^ mice expressed high levels of CD38 (Fig. 2A and 2B). The level of CD38 expression was also higher on WT Tregs than on p110δ^D910A^ Tregs from the spleen (Fig. 2C). Foxp3^−^CD4^+^ T cells of both genotypes expressed similar and low levels of CD38, suggesting that the reduced CD38 expression in p110δ^D910A^ mice is limited to Tregs.

To determine if the expression of CD38 on p110δ^D910A^ Treg is governed by signaling within the T cells themselves or by an extrinsic factor, competitive bone-marrow chimeras were generated. In these experiments, we mixed WT or p110δ^D910A^ (both CD45.2^+^) bone marrow cells with bone marrow cells from B6.SJL CD45.1^+^ congenic mice and injected these mixture into lethally irradiated RAG2^−/−^ mice to generate WT∶B6.SJL and p110δ^D910A^∶B6.SJL chimeras, respectively. After eight weeks, the expression of CD38 on CD4^+^Foxp3^+^ Treg from the spleens of these chimeric mice was determined. In the spleens of WT∶B6.SJL chimeras, CD4^+^Foxp3^+^ Treg cells from both donors showed identical expression of CD38 (Fig. 2D and 2E). However, in p110δ^D910A^∶B6.SJL chimeras, the CD45.2^+^ cells showed a lower expression of CD38 (Figs. 2D and 2E). The failure to express substantial amounts of CD38 is therefore due to an intrinsic defect within the p110δ^D910A^ Tregs.

### CD38 defines a highly suppressive subset of Tregs

**Figure 3:**
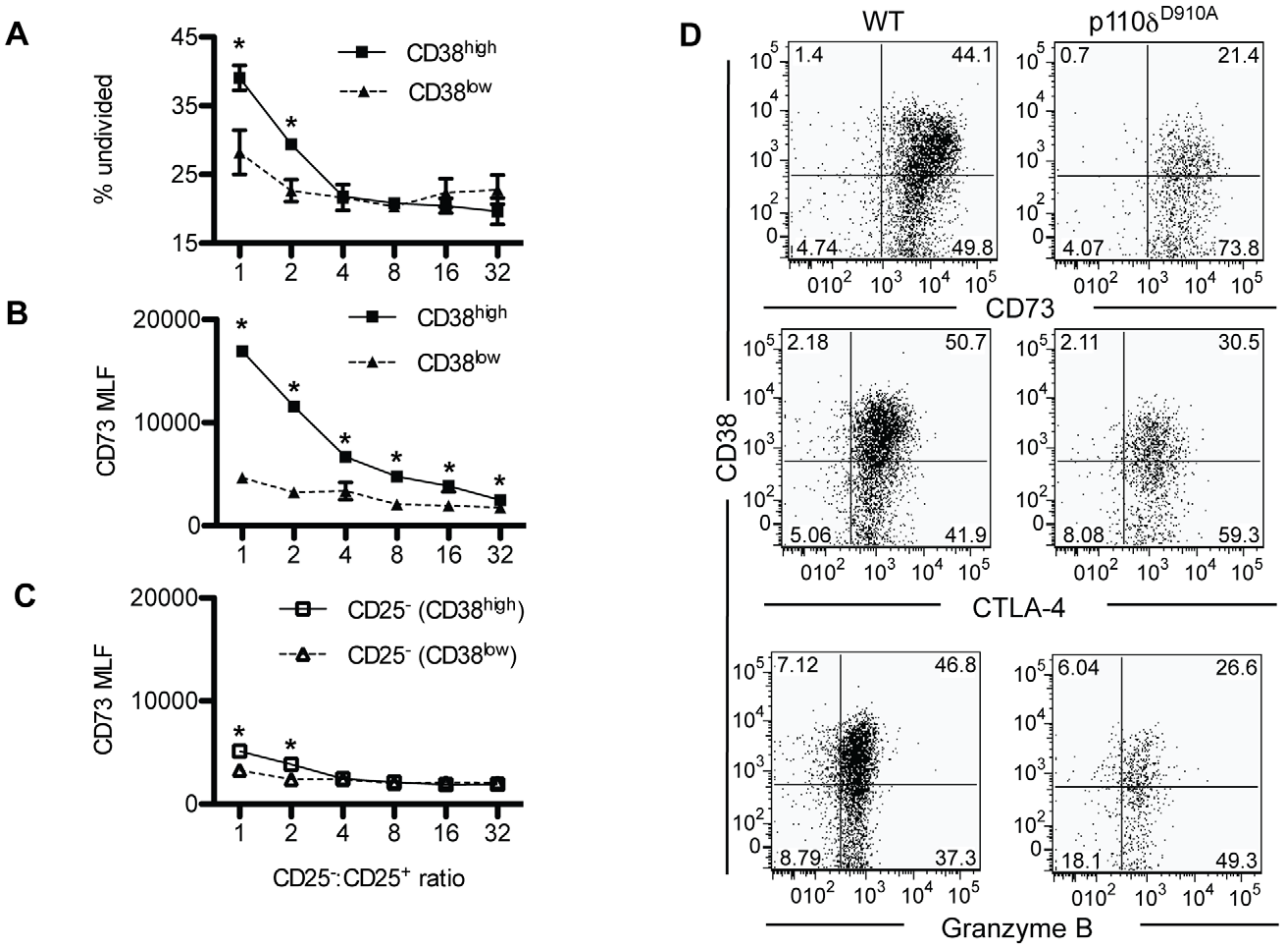
*In vitro* suppression of T cell proliferation by CD38^high^ and CD38^low^ Treg. A. Proportion of undivided CD4^+^CD25^−^ after three days culture with CD38^high^ or CD38^low^ CD4^+^CD25^+^ Tregs. B. Expression of CD73 by CD38^high^ and CD38^low^ Tregs after suppression. C. Expression of CD73 by CD4^+^CD25^−^ cells after suppression by either CD38^high^ or CD38^low^ Treg. D. Expression of CD38 versus CD73, CTLA-4 and Granzyme B on CD4^+^CD25^+^Foxp3^+^ cells from WT and p110δ^D910A^ Treg cells.

To determine if the level of CD38 expression correlates with suppressive ability, CD4^+^CD25^+^CD38^high^ or CD4^+^CD25^+^CD38^low^ cells were sorted from B6 mice and co-cultured with CFSE labeled B6.SJL responder cells. After three days CD38^high^ Treg cells suppressed CD45.1^+^ responder cells proliferation better than did CD38^low^ Treg (Fig. 3A). No difference in the survival of CD38^low^ Treg at the end of the experiment was observed (data not shown), suggesting that equivalent numbers of Tregs were available for suppression throughout the experiment. CD38 ligation has been described to result in selective induction of CD73 expression [37], which in turn is critical for Treg-mediated suppression [11]. In co-culture experiments, CD38^high^ Tregs upregulated CD73 at higher Treg∶T ratios but did not affect the CD4^+^CD25^−^ responder cells (Fig. 3B and 3C). We also confirmed that the level of CD73 expression was higher on the CD38^high^ subset of Tregs than on the CD38^low^ subset directly ex vivo (Fig. 3C). This effect appears specific to CD73, as expression of CTLA-4 and Granzyme B was similar on CD38^high^ and CD38^low^ cells (Fig. 3D). Hence, we have defined two new distinct sub populations of Tregs, one that is CD38^high^ and able to up-regulate CD73 and suppress the responses of other T cells and CD38^low^ Treg which cannot upregulate CD73.

### Reduced expression of CD38 on p110δ^D910A^ Treg is insufficient to cause altered Treg development

**Figure 4:**
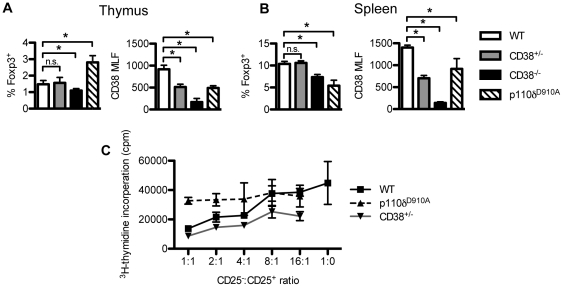
Intact development and function of Treg development in CD38^+/−^ mice. Percentage of Foxp3^+^ cells and mean linear fluorescence (MLF) of anti-CD38 antibody staining on CD4^+^Foxp3^+^ in the thymus (A) and spleen (B) of WT, CD38^+/−^, CD38^−/−^ and p110δ^D910A^ mice. (C) Comparison of suppression of CD4^+^CD25^−^ proliferation by WT, p110δ^D910A^ or CD38^+/−^ Tregs.

CD38^−/−^ mice have reduced numbers of CD4^+^CD25^+^Foxp3^+^ cells in the spleen, as previously reported [36] (Fig. 4B). However, the absence of active p110δ lead to a reduction, but not complete loss of CD38 in Tregs (Fig. 2C), similar to the expression of CD38 in thymus and spleen of CD38^+/−^ heterozygous mice (fig 3A). To determine if altered number of Tregs in p110δ^D910A^ mice is related to their lower expression of CD38, we compared the proportions of Tregs in the thymus and spleen of WT, CD38^+/−^, CD38^−/−^ and p110δ^D910A^ mice (Fig 4A and 4B). Unlike in p110δ^D910A^ mice, the proportions of Tregs in the spleen and thymus were not altered in CD38^+/−^ mice. Therefore, altered CD38 expression on p110δ^D910A^ Tregs is not sufficient to explain the reductions in the numbers or function of Treg cells in p110δ^D910A^ mice. Moreover, CD38^+/−^ Tregs suppressed the proliferation of responder cells as well as WT Treg did, under conditions where p110δ^D910A^ Treg showed minimal suppression (Fig. 4C).

### CD38 is induced by ATRA on Treg cells

**Figure 5:**
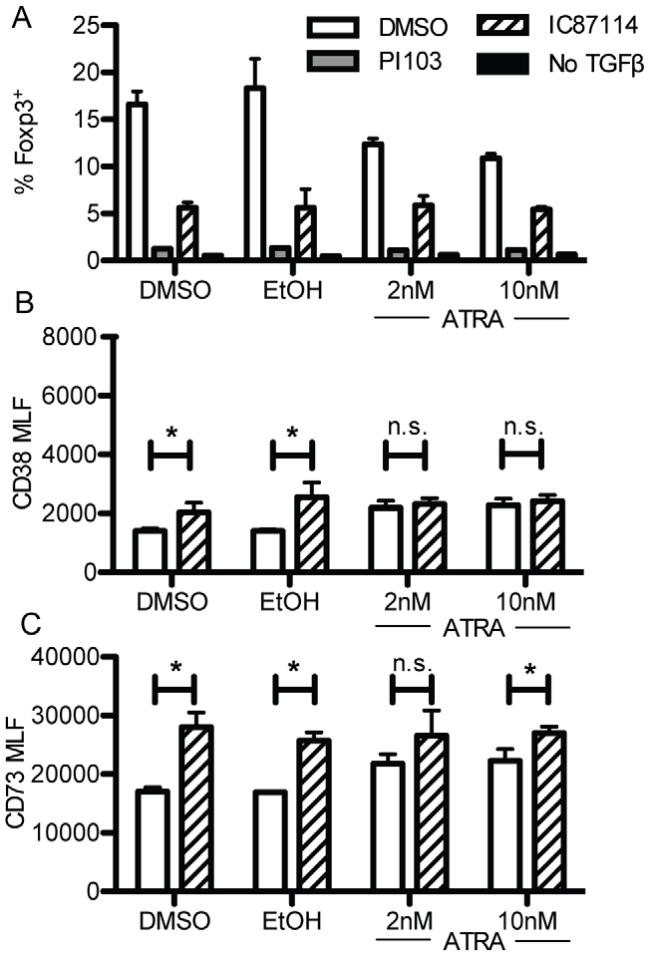
Induction of CD38 on induced Treg cells is mildly enhanced by IC87114 and ATRA. TGF-β-dependent conversion of CD4^+^CD25^−^ cells in presence of different concentrations of ATRA and the PI3K inhibitors IC87114 or PI103. (A) Percentage of Foxp3^+^ cells after 3 days of stimulation with anti-CD3, TGF-β and indicated drugs. (B) Mean expression of CD38 on Foxp3^+^ cells induced in A. (C) Mean expression of CD73 on Foxp3^+^ cells induced in A.

Expression of CD38 is regulated by several factors including the retinoic acid receptor which binds to the CD38 promoter [38], [39]. Since all-trans retinoic acid (ATRA) has been described to play a role on Treg biology [40], we investigated its role in CD38 expression on regulatory T cells. Tregs were induced from CD4^+^CD25^−^Foxp3^−^ T cells by stimulating the cells with anti-CD3 in the presence of TGF-β with or without ATRA. The pan-PI3K inhibitor PI103, the p110δ-specific inhibitor IC87114 or DMSO vehicle control were also added to mimic the p110δ^D910A^ genotype pharmacologically. After three days of culture, TGF-β induced Foxp3 which was blocked by the PI3K inhibitors. PI103 had a more potent effect suggesting that PI3K isoforms other than p110δ contribute to Treg formation *in vitro*. ATRA had little effect on the proportion of Foxp3^+^ cells produced either on its own or in combination with PI103 or IC87114 (Fig. 5A). We next examined the expression of CD38 and CD73 on TGF-β induced Treg cells. ATRA had no effect on the proportion of Foxp3^+^ Treg developing, but enhanced the level of CD38 to the same level found in IC87114-inhibited T cells (Fig 5B). Hence, ATRA enhances CD38 expression on Treg. Curiously, IC87114 blocked Treg induction, yet enhanced CD38 and CD73 expression on the few Tregs that were induced (Fig. 5C). These results indicate that ATRA may contribute to increased CD38 and CD73 expression independently of p110δ activity on Treg. Moreover, p110δ activity appears not to be required CD38 expression on induced Treg.

## Discussion

The mechanism that underlies the impaired capacity of p110δ^D910A^ Treg is incompletely understood. We therefore sought to identify other genes that may help explain the reduced function of p110δ^D910A^ Treg. We found 83 probe sets that showed greater than two-fold increased expression in p110δ^D910A^ Treg and 42 probe sets that showed reduced expression. Given the importance of Foxo transcription factors for Treg development and the key role p110δ plays in the regulation of [30], we anticipated that Foxo target genes would be over-represented among the genes that were expressed at higher levels in p110δ^D910A^ Tregs. However, there were no candidates among the over expressed genes that are known to us to be Foxo targets. One possibility is that such genes might be more readily identified in arrays from acutely activated Tregs in which Foxo may be more completely excluded from the nucleus. Another consideration is that while PI3Ks are important regulators of Foxo transcription factors, many downstream signaling pathways controlled by PI3Ks regulate post-transcriptional, metabolic, cytoskeletal and other relevant cellular events [41]. Hence, inhibition of PI3K signaling can have profound effects on cellular function without directly affecting the rates of transcription of selected genes. It follows that some of the changes we did note in mRNA abundance may not reflect direct transcriptional control by Akt, Foxo and other proteins immediately linked to PI3K activity, but rather reflect broader changes in cell biology with knock-on effects on the transcription of selected genes.

The expression of CD38 by p110δ^D910A^ Tregs was shown to be reduced by the gene arrays, qRT-PCR and most importantly, at the level of protein expression. CD38 is a transmembrane glycoprotein which has both enzymatic activity and which acts as a receptor. The extracellular domain of CD38 acts on NAD(P) to generate a wide range of products including cyclic adenosine diphosphate ribose (cADPR), nicotinic acid-adenine dinucleotide phosphate (NAADP) and nicotinamide [42], [43], [44]. cADPR can act on ryanodine receptors on the endoplasmic reticulum to stimulate Ca^2+^ release; however its exact role is uncertain as it is generated extracellularly and cannot readily penetrate the membrane. It has been suggested previously that the major role of CD38 is to limit the availability of NAD^+^ to the mono-ADP-ribosyltransferase ART2 [45]. ART2, in the presence of NAD^+^, ribosylates P2X7 resulting in the rapid apoptosis of CD4^+^CD25^+^ cells [46], [47], [48]. CD38^−/−^ mice show enhanced development of autoimmune diabetes in NOD/Lt and this effect is dependent on expression of ART2 [36]. We were unable to isolate Tregs from CD38^−/−^ mice, presumably because the cells die during the preparation of the cell suspensions [48]. However, we observed no decrease in the survival of sorted CD38^low^ cells over CD38^high^ cells, despite the reduced viability of CD38^−/−^ Treg *in vitro*. Thus, minimal levels of CD38 expression appear to be sufficient to prevent apoptosis.

CD31 (also known as PECAM) is a ligand for CD38 and in a parallel study, we found that CD31^−/−^ Treg show reduced suppressive ability, suggesting that a potentially complex role for CD31–CD38 interactions in Treg function [49], . CD38 is found within rafts in close association with LAT and the intracellular domain can directly bind the SH2 domain of Lck [51]. Ligation of CD38 results in translocation of several important signaling proteins to those rafts, including SOS and p85. Hence, the possibility that CD38 transmits signals in Treg also needs to be considered.

Our results suggest that expression of CD38 on Treg cells is controlled, probably indirectly, by p110δ during the development of Treg in the thymus. Since Treg suppression potential correlated with CD38 expression levels in WT cells, but was unaffected on CD38^+/−^ Treg, which expressed lower levels of CD38, we speculate that CD38 expression per se may not directly affect the potency of Tregs, but rather correlates with a yet-unidentified factor which promotes Treg-mediated suppression. Further research is required to fully understand the molecular basis for the impaired suppressive activity of p110δ^D910A^ Tregs. Nevertheless, our work identifies CD38 as a marker that may be used to purify highly suppressive Treg. This may be of use in clinical preparation of human Tregs, for instance, where isolation of Treg is made more challenging by the lack of cell surface proteins that unequivocally identify Tregs. Moreover, further mining of the dataset presented here may provide new leads in efforts to map genes that facilitate Treg-mediated suppression.

PI3K inhibitors are currently being developed for a variety of indications. Indeed, clinical trials have recently been initiated with CAL-101, an inhibitor that selectively blocks p110δ activation [52]. A potential consideration has been that inhibition of Treg may be detrimental for the treatment of autoimmune diseases although perhaps beneficial in the context of anti-cancer therapies. If the impaired ability of p110δ deficient Tregs to suppress is primarily due to a developmental lesion (as evidenced by the CD38^low^ phenotype originating in the thymus), then the current results leave open the possibility that acute therapeutic inhibition of p110δ will not necessarily have an adverse effect on Treg function.

## Supporting Information

Table S1 **qRT-PCR primers used in this study.** The primers listed were used to determine expression of genes identified to be increased or decreased more than two fold by gene array analysis.
(DOC)

Table S2 **Genes that were increased or decreased more than two-fold.** The genes listed in this table were increased or decreased at least two fold in p110δ^D910A^ Tregs relative to WT Tregs. Column 1 shows the Affymetrix gene probe identifier. Column 2 shows the p value (only genes with p<0.01 are included in this table). The genes are sorted according to the log difference in gene expression. Genes that were more highly expressed in p110δ^D910A^ Tregds are listed in green and those that were expressed at lower levels in red.
(XLS)
