# Supplementary material for: The PI3K p110δ Regulates Expression of CD38 on Regulatory T Cells
Source: PLoS One. 2011 Mar 1;6(3):e17359. doi: 10.1371/journal.pone.0017359 (PMC3046981; doi:10.1371/journal.pone.0017359)
Supplement: Table S1 — qRT-PCR primers used in this study. The primers listed were used to determine expression of genes identified to be increased or decreased more than two fold by gene array analysis. (DOC) [file pone.0017359.s001.doc]

Table S1. qRT-PCR primers used in this study

| Gene | Forward Primer | Reverse Primer |
| --- | --- | --- |
| Stard5 | AGAAGTGTCGGGAAGGCAATG | AACTGGCTTTATGCAATCCCAC |
| IL4 | CCATGCTTGAAGAAGAACTCTAGTGT | AGTGATGTGGACTTGGACTCATTC |
| Btbd11 | CCCCTCTCATGGAGTGGATC | AGAAGCACAAAAGCAGTCGTC |
| Plac8 | CAGCCTGTGTGATTGCTTCAG | CAGACAACACTCATTCATGTCAGC |
| Mpeg8 | CTGGATGATAATAGCGTGTGC | CAAGACAGGTAGTTTCAGGGC |
| Atp6v0d2 | AAGCCAGCCTCCTAACTCAGC | TTAGCCAGGAAGTTGCCATAGTC |
| Idb2 | GAACACGGACATCAGCATCCT | GCTATCATTCGACATAAGCTCAGAAG |
| Itgb1 | AGACTTCCGCATTGGCTTTGG | GGCTGGTGCAGTTTTGTTCAC |
| Lag3 | CTGGGACTGCTTTGGGAAG | GGTTGATGTTGCCAGATAACCC |
| Ccdc28b | AGCAGCGAGCCAAGTTCAAG | TCTGTCAGGAAGGAGTGTTGCA |
| Faim3 | ATGACGATGGAATCTATGCCTGT | AATCTGTGCAACCATCTTGGC |
| Irf7 | ATCCAGTTGATCCGCATAAGG | GCATTGCTGAGGCTCACTTC |
| Oas2 | ATGAAAGTGTCGAGTTCGATGTC | GCTCAATGAGATCCTTGTAGGC |
| Ehd4 | GCACAGGACCTCTTCCGAGAC | GCTGATGATGTAGGCGTGGAC |
| Lisch7 | CACCTGCTGCTGCTATGTCAG | AGATGCTTGGCACACCTGAG |
| Irf4 | CTCAGAGACAGAGGAAGCTCATCAC | GCGGTGGTAATCTGGAGTGG |
| Derl3 | TTCTCTTCGGTGGTGTTCTTATGAC | CTGCGACGGCTCCATACATAG |
| Slfn1 | CTAAATGCAGGAGGGATCACAC | AGAGCACACAGAGCTTTTGTAATG |
| Sh3gl3 | TAGAGGCGGCATTAGACTATCACAG | CATTGGCATCGGTGGAACTC |
| Gzmk | TGGCTGGCGTTTATATGTCTTC | GCTGCGGTACTGGATGGACG |
| Ly108 | CGCCTGTCAGAGGATGGTCT | GCAGACTCTCCTAGAACGCCAT |
| Il1r2 | AGTGCAGCAAGACTCTGGTACCTA | AGTTCCACAGACATTTGCTCACA |
| Sp6 | ACACCGGGACCAAGAAGTTC | CGCCTTCGTGGGTTTTCATG |
| Icos | TGAAGCCGTACTTCTGCCATG | CGCATTTTTAACTGCTGGACAG |
| Itk | TTCCTCCTACTCCTGAAGACAACA | GGTAGTCGTACAAGGCAATGACC |
| Kif1b | AGGACCTTCTTCGTGCTCAGG | AGCATGAAGATGGAGATGAAGTGAG |
| Plekho1 | TGAAGAGAAGGAGTCATGGATCAAC | GCTGTCCTCCTCAACGGTGA |
| Socs5 | GACGGCTTAGTATCGAAGAA | GCTTATACAATGGGTTGACC |
| Hipk2 | GACAACCGTACCGAGTGAAGG | TCAGCAATGACACAACCAAGG |
| Nfll3 | GGGAATTAGCAAACTTATCTGC | TTACCTGGAGTCCGAAGCCGAG |
| Sytl2 | GAGCGATGACAGAGAAACAGAC | AAGATTGGTTAATGAGCTGGGG |
| Tcf7 | AAGAAGAAGAGGCGGTCAAGG | ACTGTCATCGGAAGGAACGG |
| Prdm1 | GAACCTGCTTTTCAAGTATGCTG | AGTGTAGACTTCACCGATGAGG |
| Lrpap1 | CTGAAGGTGGAAGGCTTGGAT | GGATGACGTTGAGGTTGTGGA |
| Tiam1 | CATGCAAGGCAGAAGAGCAA | TGGATCTCCTGGACAAGAGGTC |
| Prdm16 | CCACCAGATGTCTCACGACAGT | ATGTGACGCTGGAGGTTGCT |
| Nrip1 | AGACCAGAACTTTAACCTCTCGG | CGATGGAATCAGACAGCCTCT |
| Adk | GAGCAGAACGAGCAGCCAAC | CAGTTCCGCTCCAGGTCAAG |
| CD226 | GAAGAGACGGAGACAGGTGAGAA | GAAGTAGGACTTCTGCAGTTGGTG |
| Lad1 | CAGCATGTCGGTCAGCAGAA | GCTCCTCTTCGTCCTCCAAAG |
| Gucy1a3 | AAGACAAGCCGCAACAGAGTC | GCAAGGTTCAGTCGCTCAC |
| Ccr6 | CCTGGGCAACATTATGGTGGTG | CAGAACGGTAGGGTGAGGACA |
| Itgae | CACTAAGACCGCCTCAGCCA | AATATGTCACCATCAGTAAGTACCACCA |
| Gnaq | CCAGGAGTGCTACGACAGACG | GCTACACGGTCCAAGTCATTCAG |
| Penk1 | TGCGCTAAATGCAGCTACCG | TCCCAGATTTTGAAAGAAGGCAG |
| Sh2bgrl2 | GTCTTCGTCGCCTCATGCTC | TCGAACTCTATCTTGTTGGCTTCC |
| Tnfrsf9 | AGAAGGACGTGGTGTGTGGAC | CGCCAGGAACAAGGTAAGGA |
| CD38 | GGAGAGCCTACCACGAAGCA | TGTACTCAGTATCTCCTGGCAGTTCT |
| Rab6b | CCAGCTCATCCGAGACTCCA | AGAAGTCTGCTGGAAGGAGTTGAG |
